# Influenza Virus Infection of Human Lymphocytes Occurs in the Immune Cell Cluster of the Developing Antiviral Response

**DOI:** 10.3390/v10080420

**Published:** 2018-08-10

**Authors:** David J. Mock, Mark W. Frampton, Joan E. Nichols, Frank M. Domurat, Denise J. Signs, Norbert J. Roberts

**Affiliations:** 1 Department of Medicine, School of Medicine, University of Rochester, Rochester, NY 14642, USA; david_mock@urmc.rochester.edu (D.J.M.); mark_frampton@urmc.rochester.edu (M.W.F.); fdomurat@gmail.com (F.M.D.); bugsrus254@aol.com (D.J.S.); 2 Department of Internal Medicine, University of Texas Medical Branch, Galveston, TX 77555, USA; jnichols@utmb.edu; 3 Division of Infectious Diseases and Immunology, Department of Medicine, New York University School of Medicine, New York, NY 10016, USA

**Keywords:** influenza virus, human monocytes, human macrophages, human lymphocytes, immune cell clusters, alveolar lymphocytes

## Abstract

Monocytes-macrophages and lymphocytes are recruited to the respiratory tract in response to influenza virus challenge and are exposed to the virus during the establishment of immune defenses. The susceptibility of human lymphocytes to infection was assessed. The presence of monocytes-macrophages was required to attain infection of both resting and proliferating lymphocytes. Lymphocyte infection occurred in the context of immune cell clusters and was blocked by the addition of anti-intercellular adhesion molecule-1 (ICAM-1) antibody to prevent cell clustering. Both peripheral blood-derived and bronchoalveolar lymphocytes were susceptible to infection. Both CD4^+^ and CD8^+^ T lymphocytes were susceptible to influenza virus infection, and the infected CD4^+^ and CD8^+^ lymphocytes served as infectious foci for other nonpermissive or even virus-permissive cells. These data show that monocytes-macrophages and both CD4^+^ and CD8^+^ lymphocytes can become infected during the course of an immune response to influenza virus challenge. The described leukocyte interactions during infection may play an important role in the development of effective anti-influenza responses.

## 1. Introduction

Murine models have been used to demonstrate the rapid and substantial recruitment of peripheral blood mononuclear cells (PBMC), both monocytes-macrophages and lymphocytes, to the lung after influenza virus challenge [1,2,3,4]. These recruited cells play important roles in defense against and recovery from the virus infection [2,5,6], demonstrated by studies using adoptive transfer [7,8] or host immunosuppression [9,10,11]. Notably, recruited human PBMC may themselves become infected by influenza virus in the context of developing the immune defense response in the respiratory tract [12].

The immunological synapse is an early and key feature of the host’s response to pathogen challenge [13,14]. Direct physical interaction between monocytes-macrophages and T lymphocytes has been reported to occur within hours after exposure of PBMC to mitogens or antigens, including influenza virus [15,16,17,18]. Exposure to influenza virus results in enhanced expression of the lymphocyte function-associated antigen-1 (LFA-1) and its ligand intercellular adhesion molecule-1 (ICAM-1) by both monocytes-macrophages and lymphocytes [19]. In earlier studies, the presence of monocytes-macrophages was shown to be necessary for the infection of human lymphocytes by influenza A, including H1N1, H2N2, and H3N2 strains of the virus. The need for monocytes-macrophages was not offset by factors derived from those cells, exogenous enzymes, or high multiplicities of infection [20]. The infection of both monocytes-macrophages and lymphocytes was abortive, with evidence of virus-directed protein synthesis, but without the release of free, infectious viral progeny [21,22,23]. Monocyte-macrophage-dependent infection of lymphocytes might be expected to occur during immune cell cluster formation induced either by the influenza virus itself or by the preceding antigen or mitogen stimulation [15,16,17,24].

The current studies were designed to examine human PBMC cultures for such an association of immune cell clusters with the process of influenza virus infection. We determined the susceptibility of CD4^+^ and CD8^+^ subsets of T lymphocytes to infection, and whether monocytes-macrophages were required for the uptake of influenza virus by lymphocytes, or merely for the activation of the lymphocytes to a state (similar to that of mitogen-stimulated cells) that supported the synthesis of viral proteins after independent uptake of the virus by those cells. The results indicate that macrophage-to-lymphocyte transfer of influenza virus occurs in the context of the immune cell cluster that is a critical component of the developing antiviral host response.

## 2. Materials and Methods

### 2.1. Cell Sources and Culture Conditions

PBMC were obtained from the peripheral blood of healthy volunteers by Ficoll-Hypaque sedimentation [25]. Informed consent for withdrawal of blood was obtained from all donors. Donors of peripheral blood only ranged in age from 18 to 45 years. Donors of both bronchoalveolar lavage (BAL) cells and peripheral blood-derived cells were healthy men and women between the ages of 20 and 40 who met the following requirements: no pulmonary disease by history and physical examination, no present or past history of smoking, absence of upper respiratory illness for at least six weeks prior to study, and normal spirometry. Informed written consent was obtained from the subjects for collection of autologous BAL and peripheral blood cells. Informed oral consent was obtained for collection of peripheral blood cells only from a donor. The studies and methods of consent were approved by the Institutional Review Boards for Human Subjects Research of the University of Rochester and the University of Texas Medical Branch. 

Bronchoalveolar lavage was performed as described previously [26], using a fiberoptic bronchoscope (Pentax FB-19H, outer diameter 6.3 mm). Lavage cell viability exceeded 95%. Differential counts were performed by assessing 500–1000 cells on a cytospin smear stained with Diff-Quick stain (American Scientific Products, McGraw, IL, USA). Alveolar cells were 89.2 ± 4.6% (mean ± SD) alveolar macrophages by morphology, with the remainder of cells predominantly lymphocytes. 

Equal numbers of male and female subjects were used as volunteer donors. It was expected that all donors had experienced past in vivo exposure to influenza virus. All experiments used concomitant assays of autologous cell preparations. Viability of cells was determined by ability to exclude trypan blue dye, and purity was determined by staining for nonspecific esterase activity [27] as well as phenotyping by flow cytometry. The PBMC were either exposed to infectious influenza virus immediately after collection or were separated into PBMC subpopulations before exposure to virus, as described below. The PBMC were cultured at 37 °C in medium 199 (M199) with 10% heat-inactivated fetal calf serum, except during the one-hour exposures to infectious influenza virus in serum-free medium [27,28].

### 2.2. Purification of Peripheral Blood Monocytes-Macrophages, Total Lymphocytes, and Subsets of Lymphocytes

Purified lymphocytes, moderately or severely depleted of monocytes-macrophages, were obtained from PBMC by countercurrent centrifugal elutriation using previously described techniques with minor modifications [20,29,30,31]. Elutriation was conducted at 4 °C using a Beckman J2-21 centrifuge with a JE-6 elutriator rotor and Multiperplex pump with fine velocity control (LKB Instruments), with rotor and flow speeds based on earlier studies [20,31]. The lymphocytes obtained by elutriation were shown to have >99.5% purity and >97% viability (Figure S1). Purified monocytes and monocyte-derived macrophages were obtained by adherence of PBMC in plastic culture dishes for 1–7 days, followed by extensive washing to remove nonadherent cells, and scraping and collection of adherent cells with a rubber policeman [27,28]. In a subset of experiments, monocytes were obtained from the “blow-out” fraction of elutriation (by turning off the rotor after elutriation of lymphocytes). There were no differences in results when comparing monocytes-macrophages obtained by adherence with those obtained by elutriation.

Total T lymphocytes were further separated into CD4^+^ and CD8^+^ lymphocyte subpopulations by indirect panning [32], using either anti-CD8 or anti-CD4 antibody (Becton Dickinson, Mountain View, CA, USA). Both captured (positively selected) CD4^+^ or CD8^+^ cells and, in a subset of experiments, nonadherent (negatively selected) cells were obtained. The negatively selected CD4^+^ and CD8^+^ cells (obtained by panning for elimination of the alternate subset) provided viral protein synthesis data similar to that presented in Results for positively selected cells. However, the purity (>90%) of the negatively selected subset populations was less than that of positively selected lymphocytes (although free of monocytes-macrophages), and the data for the latter cells only are presented in Results. The positively selected highly purified CD4^+^ and CD8^+^ subsets of T lymphocytes were free of detectable monocytes-macrophages (Figure S2).

The purity of cells used for measurements of viral protein synthesis was confirmed by immunofluorescent staining and flow cytometry analysis, as well as by staining for nonspecific esterase activity. Elutriated purified resting lymphocytes were free of monocytes-macrophages (none detectable; at least 10,000 cells/sample analyzed), and even the proliferating lymphocyte preparations (large-cell fractions in experiments described below) contained <0.5% monocytes-macrophages, which in concomitant control determinations, could not account for detectable viral protein synthesis upon subsequent examination. Monocytes-macrophages, obtained by collection of adherent cells after extensive washing, were >97% pure. For all assays, control experiments determined that the maximal number of possible contaminating cells (e.g., monocytes-macrophages in lymphocyte preparations at the limits of detection) could not account for the results.

### 2.3. Exposure to Infectious Influenza Virus

The PBMC, BAL cells, or purified subpopulations of PBMC were exposed to infectious influenza A/Marton/43 (H1N1) at a multiplicity of infection (MOI) of 3 for one hour at 37 °C in serum-free medium [27,28]. Purification of T lymphocytes and lymphocyte subsets was performed after exposure to influenza virus (in the presence of the adherent macrophages) to determine relative susceptibility of the different subsets of cells to virus infection. In other experiments, CD4^+^ and CD8^+^ T lymphocyte populations were first purified, layered over adherent macrophages and exposed to virus, and then collected again in order to determine intrinsic susceptibilities of the different subpopulations to infection.

In a subset of experiments, sham-exposed and influenza virus-exposed PBMC, monocytes-macrophages, or lymphocytes were treated with neuraminidase (*Clostridium*-derived, Type V, Sigma, St. Louis, MO, USA) to remove surface-bound virus [24,33]. Previous studies by others have shown that neuraminidase treatment removes adherent radiolabeled influenza virus from the cell surface [33,34]. In our own studies, flow cytometric analyses of cells that were exposed to fluorescein isothiocyanate (FITC)-labeled virus and treated similarly with neuraminidase showed that all detectable virus was removed from the cell surface [35].

In another subset of experiments, PBMC and lymphocytes, depleted of monocytes-macrophages by elutriation, were exposed to FITC-labeled influenza virus at a MOI of three [35]. The virus was labeled using described methods [35,36] and remained fully infectious [35]. Cells were collected and analyzed by flow cytometry after exposure to the FITC-labeled virus. In a series of these experiments, PBMC were treated with anti-ICAM-1 monoclonal antibody [18] (AMAC, Inc., Westbrook, ME, USA) or an isotype control antibody prior to exposure to the FITC-labeled virus.

### 2.4. Incremental PBMC Monocyte/Macrophage Depletion and Collection of Resting And Proliferating Lymphocytes

Elutriation of fresh PBMC was also used to examine both (a) the effect of varying the ratio of monocytes-macrophages to lymphocytes and (b) the effect of the proliferative state of the lymphocytes in regard to susceptibility of those cells to infection, with associated synthesis of viral proteins (Figure S3). On day zero, the PBMC were elutriated to produce three parallel populations differing only in the ratio of monocytes-macrophages to lymphocytes. Highly purified lymphocytes (<0.5% monocytes-macrophages) were obtained from fresh PBMC using the above-described elutriation protocol. In parallel arms of the experiments, aliquots of cells were collected (a) by varying the flow and rotor speeds (27 mL/min and 3400 rpm) to give cell preparations that were shown to contain 11% monocytes-macrophages, or (b) by “blowing out” the chamber (27 mL/min, rotor off) to give PBMC containing 26% monocytes-macrophages (a percentage identical to the fresh PBMC). Thus, in the latter arm, cells were put through the elutriation chamber as a procedural control rather than for the purpose of depleting or enriching subpopulations of cells. The three PBMC preparations, containing <0.5%, 11%, or 26% monocytes-macrophages, were then stimulated to proliferate by addition of phytohemagglutinin (PHA, 20 μg/mL; Difco, Detroit, MI) or heat (2 h, 56 °C)-inactivated influenza A/Marton/43 (H1N1) (viral antigen equivalent to a MOI = 1) for one hour [27,37,38]. The cells were then washed and cultured for three days. (There was no synthesis of viral proteins by PBMC exposed to the inactivated virus.) On day three, the stimulated PBMC were exposed to infectious influenza virus at a MOI of 1 or 3. The lower MOI was chosen initially, and used for most experiments, to facilitate observation of any enhanced susceptibility of the proliferating lymphocytes in the presence (or absence) of monocytes-macrophages. The data from those experiments are presented in Results (see figures). Subsequent studies using a MOI of 3 produced similar findings.

Four hours after exposure to the infectious virus, purified monocytes-macrophages (>97%) were obtained by extensive washing to remove nonadherent cells. The nonadherent cells were removed and separated by elutriation into populations (>99.5% lymphocytes) enriched for small, resting (>95% G_0_/G_1_) lymphocytes or large, proliferating (>40% S + G_2_/M) lymphocytes/lymphoblasts (Figure 4) using elutriator settings determined previously [31,39].

### 2.5. Flow Cytometry Analyses of Cell Phenotype, Cell Cycle, and Virus Uptake 

The purity of cells used for the different assays for viral infection was always confirmed by direct immunofluorescent staining and two-color flow cytometry (FACScan, Becton Dickinson) analysis [38]. Monoclonal antibodies identifying CD3^+^ T lymphocytes (anti-Leu-4), CD4^+^ cells (anti-Leu-3a), CD8^+^ cells (anti-Leu-2a), and CD14^+^ monocytes-macrophages (anti-Leu-M1), as well as isotype control antibodies (Becton Dickinson), were used in such analyses. Cells were stained using acridine orange and chromamycin for cell cycle analysis by flow cytometry [40,41]. Such analysis, and measurements of forward and side angle light scatter, were performed using a Coulter EPICS V flow cytometry system. For each flow analysis, 10,000 total cells (whether obtained from peripheral blood or bronchoalveolar lavage) were examined.

PBMC that were exposed to influenza virus that had been FITC-labeled were labeled just prior to analysis with phycoerythrin-conjugated anti-T-cell or subset (CD3, CD4, CD8) antibodies. The cells were first analyzed for green fluorescence. Ethidium bromide was then added to produce quenching of any fluorescein on virus that had not been internalized by the cells [35,42], and the cells were again analyzed within 5 min of adding the ethidium bromide to avoid cell death and uptake of the dye. All cells were maintained at 4 °C during the period of staining with ethidium bromide and flow analysis. Lymphocyte gating was accomplished by forward and side light scattering parameters [22,35].

### 2.6. Microscopic Analyses of Cell Clusters and Viral Protein Expression

The extent of cell clustering after exposure was determined by culturing cells over plastic cover slips. Cover slips from the cultures were retrieved at 1, 4, 24, and 72 h after sham-exposure or exposure to influenza virus. Cells were stained by the Diff-Quick method (Dade Diagnostics, Aquada, PR) immediately after collection of cover slips, and examined for cell clustering using a Zeiss microscope [43].

Expression of influenza virus proteins by live cells in culture was determined by indirect immunofluorescent labeling using polyclonal hemagglutinin (H1)-specific or neuraminidase (N1)-specific reference antiserum (NIH Reagents; V-314-511-517 and V-308-513-157, respectively) and examination of cells by fluorescence microscopy.

### 2.7. Analyses of Protein Synthesis

Cells were suspended in methionine-free medium and pulse-labeled with 100 μCi of ^35^S-methionine for 2 h. Cells were then washed and lysed by sodium dodecyl sulfate (SDS)-containing detergent buffer with subsequent analysis by SDS-polyacrylamide gel electrophoresis (SDS-PAGE) and autoradiography [20,44]. Lysates were also immunoprecipitated using polyclonal anti-hemagglutinin (H1), anti-neuraminidase (N1), and anti-matrix protein (V-306-510-157) antibodies (all NIH reference reagents, listed above; goat antisera), and murine monoclonal anti-nucleoprotein [20,45].

### 2.8. Northern Blot Analyses for Influenza Virus RNA

Total cellular RNA was obtained from sham-exposed and influenza virus (IAV)-exposed cells using described methods [46,47]. After prehybridization, Northern blots were hybridized with a 32P-labelled RNA strand complementary to the 1413 nucleotides of the influenza virus N1 neuraminidase positive strand (mRNA and template RNA) for 18 h. The radiolabeled RNA probe was transcribed using T7 RNA polymerase and a linearized (Hind III digest) template of a pGEM (Promega, Madison, WI, USA) plasmid containing the influenza A/WSN neuraminidase cDNA (a kind gift from Dr. Louis Markoff, NIAID). Neither the N2 neuraminidase (NA) nor input virus (negative strand) RNA was detected using this probe. Each blot was also hybridized subsequently using a probe for the human cellular gene product β-actin, derived from a cDNA cloned into the pBluescript II KS (−) vector (Stratagene, LaJolla, CA, USA), as a control for analysis of cell lysates. Densitometry was performed on the autoradiograms to compare intensities of the signals for the viral versus cellular gene products, using a Zeineh Scanning Densitometer (Biomed Instruments, Fullerton, CA, USA).

### 2.9. Infectious Focus Assay for Influenza Virus Infection

Confluent monolayers of Madin–Darby canine kidney (MDCK) cells were prepared in 24-well culture plates and maintained in minimum essential media (MEM) at 37 °C until use [48]. Sham-exposed and influenza virus-exposed leukocytes were first treated with neuraminidase as described above. The neuraminidase-treated cells were then washed extensively and diluted to 10,000, 1000, 100, and 25 cells per sample in medium containing trypsin [48]. Duplicate 200 μL samples were layered over MDCK cell monolayers and incubated at 37 °C for one hour. Cell suspensions were removed by aspiration and the MDCK cells were overlaid with 0.6% agarose and incubated at 37 °C for 48 h. The monolayers were then fixed in 10% formalin and stained with methylene blue to facilitate plaque quantitation. Based on previous studies, one plaque was assumed to be caused by one infected cell [48].

## 3. Results

### 3.1. Susceptibility of PBMC Monocyte/Macrophage and Lymphocyte Subpopulations to Influenza Virus Infection

**Figure 1:**
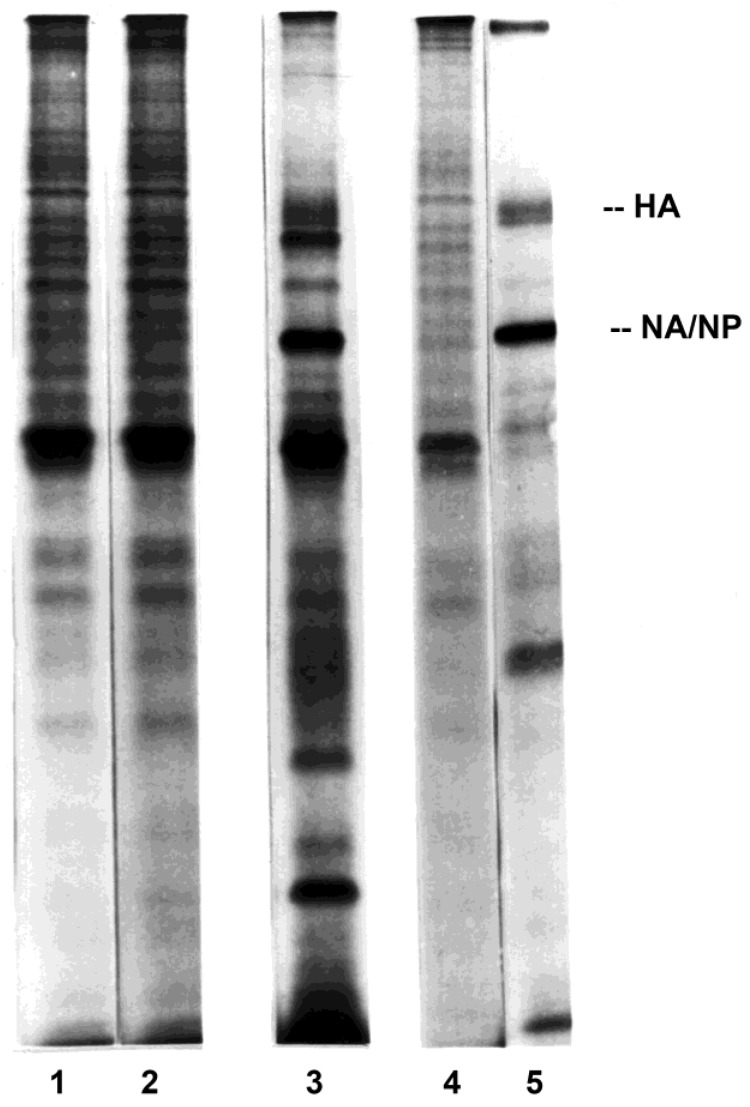
Influenza virus infection of purified PBMC subpopulations. Autoradiograms of purified human lymphocytes (lanes 1–3) or monocytes-macrophages (lanes 4 and 5) which were sham-exposed or exposed to influenza A/AA/Marton/43 H1N1 (MOI = 1.0) are shown. Lanes 1 and 2 show lysates from lymphocytes sham-exposed or exposed in the absence of monocytes-macrophages, respectively. Lane 3 shows lysate from lymphocytes exposed to virus in the presence of macrophages, and then purified by elutriation before radiolabeling. Lanes 4 and 5 show lysates from sham-exposed or exposed monocytes-macrophages, respectively. Each lane represents lysate from 2 × 10^6^ cells. HA = hemagglutinin, at the 85 kD position; NA/NP = neuraminidase/nucleoprotein, which comigrate on SDS-PAGE, at the 60 kD position.

Exposure of lymphocytes to influenza virus in the absence or presence of autologous monocytes-macrophages demonstrated a requirement for monocytes-macrophages to obtain the virus infection of autologous lymphocytes (Figure 1, lane 2 versus lane 3, respectively), as shown in earlier studies [20]. The influenza protein bands were identified by appropriate relative molecular mass marker (Mr) positions and by immunoprecipitation using monoclonal or monospecific polyclonal antibodies against viral hemagglutinin (HA), neuraminidase (NA), nucleoprotein (NP), and matrix (M) protein [20,45]. The comigrating NA/NP band and, to a lesser extent, the HA band were most readily detected in total cell lysates, and are indicated in the figures.

In preliminary experiments as well as previously published studies [20], monocytes-macrophages synthesized viral proteins over 1 to 12 h or more after exposure to the virus, with maximal synthesis from 2 to 8 h. Analyses of lysates of monocytes-macrophages and lymphocytes from cells that were pulse-labeled 4–6 h after exposure to virus best allowed a 1-h coincubation of monocytes-macrophages and lymphocytes (in the coculture arms of the experiments), followed by separation by elutriation and warming and recovery of cell protein synthesis. Similar results could be observed with pulse-labeling of the lymphocytes over the few hours preceding or following the time presented in these results, although with a reduction in absolute amounts of protein synthesis, as previously reported [20].

**Figure 2:**
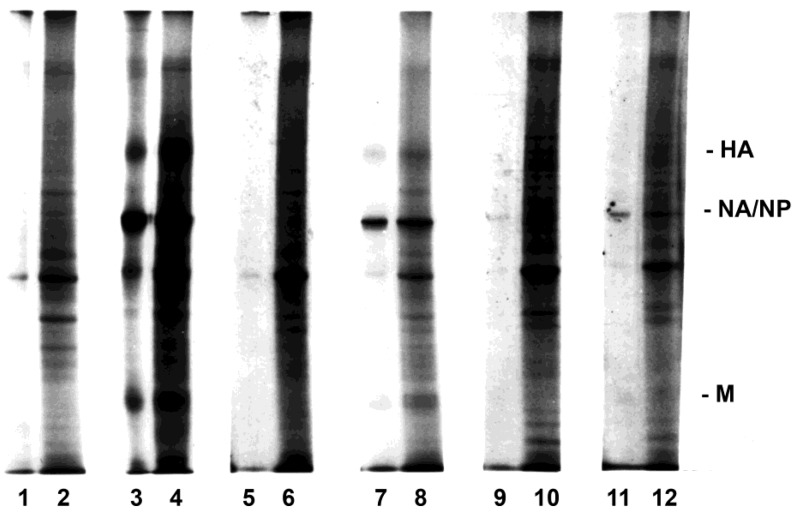
Influenza virus infection of purified PBMC subpopulations. Autoradiogram of proteins from purified human macrophages (lanes 1–4), total lymphocytes (lanes 5–8), and CD4^+^ (lanes 9 and 10) and CD8^+^ (lanes 11 and 12) T lymphocytes that were sham-exposed (controls, lanes 1, 2 and 5, 6) or exposed (all other lanes) to influenza virus are shown. Lymphocytes were exposed in the presence of macrophages (i.e., as PBMC) and then purified before radiolabeling, as described in Materials and Methods. Both immunoprecipitates using anti-hemagglutinin (HA), anti-neuraminidase (NA), anti-nucleoprotein (NP), and anti-matrix protein (M) antibodies (odd-numbered lanes) and total cell lysates (even-numbered lanes) are shown. Photographic exposure of the autoradiogram for lanes 9 to 12 was prolonged relative to the other lanes of the gel to permit illustration of the less intense bands of those lysates.

Studies were performed to determine the intrinsic and relative susceptibilities of different T lymphocytes which might interact with monocytes-macrophages and become infected, by measuring the synthesis of viral proteins. Initially, T cell subsets were first purified, then exposed to virus in the presence of macrophages (but absence of other lymphocytes), and again retrieved and radiolabeled. In such experiments, both CD4^+^ and CD8^+^ T lymphocytes were infected, as determined by the synthesis of viral proteins. In the next series of experiments, PBMC were exposed to the virus and subsequently separated into subpopulations for analyses of relative susceptibilities of the cell subpopulations. Total lymphocytes (Figure 2, lanes 7 and 8) and CD4^+^ (Figure 2, lanes 9 and 10) and CD8^+^ (Figure 2, lanes 11 and 12) T lymphocytes were susceptible to influenza virus infection when exposed in the presence of macrophages. Synthesis of viral proteins was faint due to the small percentage of lymphocytes infected, as documented later in Results, but was clearly detectable on the original gels of the experiments (such as the representative example in Figure 2), in which lymphocytes were exposed to virus (in the presence of macrophages) before purification by elutriation or by panning for subsets. Both CD4^+^ and CD8^+^ T lymphocytes demonstrated viral protein synthesis, with easier detection of viral proteins in lysates of CD8^+^ cells than CD4^+^ cells (as illustrated in Figure 2) in all but one of many experiments. The most easily detected synthesis was that of the influenza neuraminidase and the nucleoprotein, which comigrate on SDS-PAGE of the lysates.

### 3.2. Influenza Virus Neuraminidase Transcription

**Figure 3:**
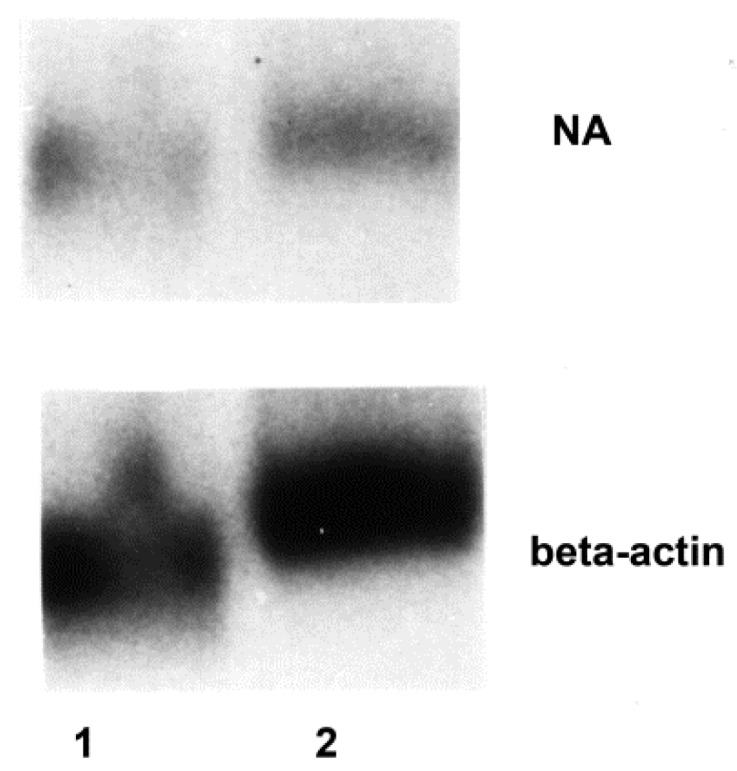
Influenza virus infection of purified lymphocyte subpopulations. Northern blot autoradiograms of lysates of CD4^+^ (lane 1) and CD8^+^ (lane 2) T lymphocytes are shown. The cells were exposed to influenza virus in the presence of monocytes-macrophages (that is, as PBMC) and then purified as described in Materials and Methods. Lysates were probed for influenza virus neuraminidase (NA) and the same lanes were probed subsequently for β-actin.

Lysates from purified lymphocytes exposed to virus in the presence of macrophages, but not from lymphocytes exposed in their absence, contained detectable neuraminidase RNA. Neuraminidase RNA could be detected in lysates of both CD4^+^ and CD8^+^ T lymphocytes when the cells were exposed to virus as PBMC (before separation for analysis; Figure 3). In contrast to the data regarding viral protein synthesis, transcription of the neuraminidase RNA appeared to be equivalent in CD4^+^ and CD8^+^ lymphocytes when the signal for the neuraminidase on the Northern blots was expressed in relationship to the signal for β-actin; the mean ratios of signals by densitometry were 1.80 and 1.86, respectively, based on several experiments. The data suggested that monocytes-macrophages were required for the transcription as well as translation of influenza virus gene products by lymphocytes.

### 3.3. Lymphocyte Proliferation and Influenza Virus Infection

**Figure 4:**
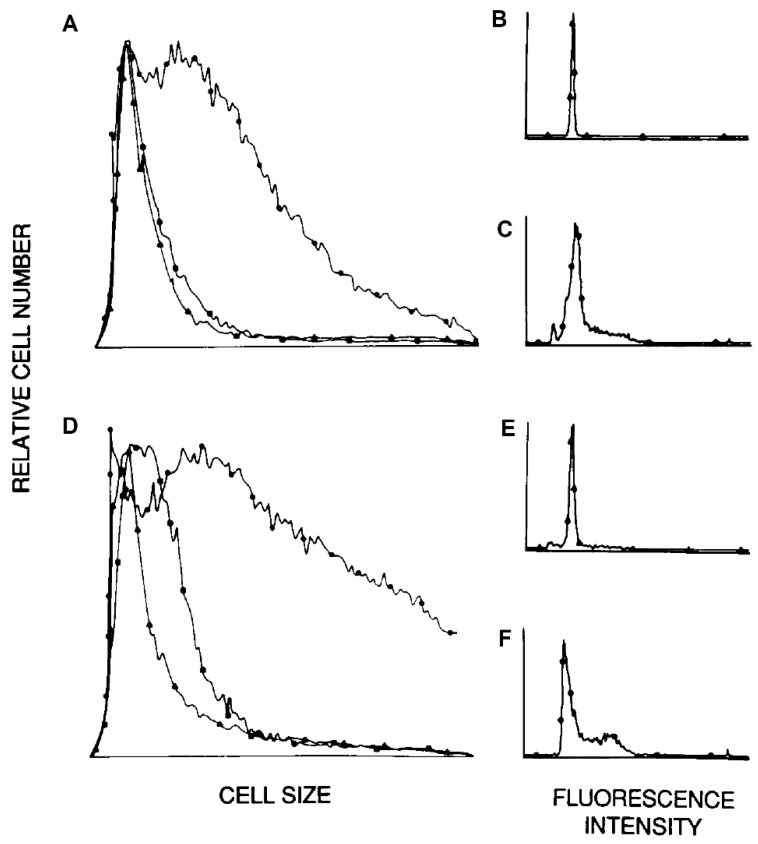
Cell size distributions (**A**,**D**) and cell cycle analyses (**B**–**F**) of human lymphocytes cultured for three days after stimulation with inactivated influenza virus in the presence of <0.5% (**A**–**C**) or 26% (**D**–**F**) monocytes-macrophages (see protocol in Figure S3). Cells were then exposed to infectious influenza virus and analyzed and separated by elutriation into fractions enriched for small (▲ in (**A**,**D**)), resting (**B**,**E**) lymphocytes, and large (●, **A**,**D**), proliferating (**C**,**F**) lymphocytes. The horizontal axes of **A** and **D** indicate linear increments in size from 0 to 1220 μ^3^. Intermediate results were obtained using lymphocytes cultured in the presence of 11% monocytes-macrophages. Intermediate fractions of lymphocytes from each monocyte-macrophage-lymphocyte coculture, obtained immediately after changing elutriator settings and containing substantial mixtures of resting and proliferating lymphocytes, were discarded without further analysis.

**Figure 5:**
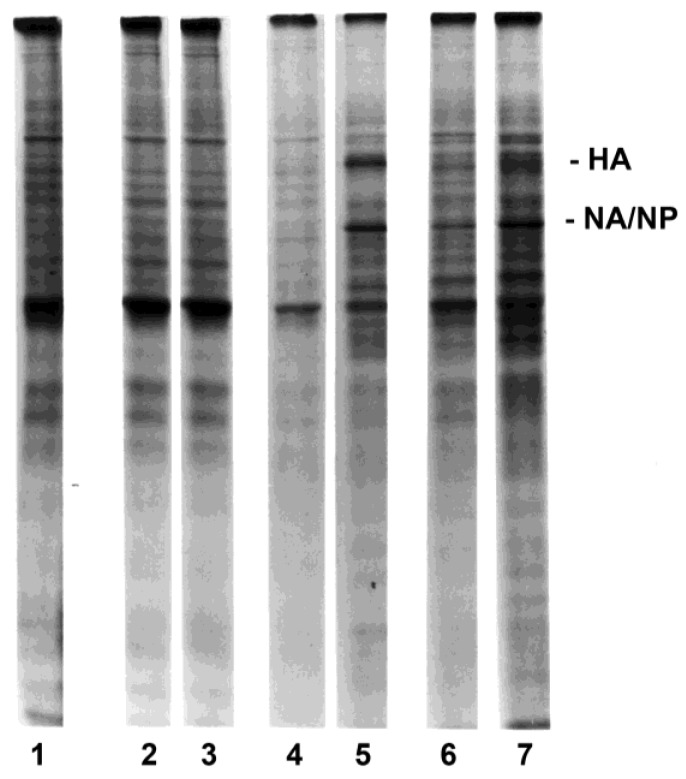
Influenza virus infection of purified resting and proliferating lymphocytes. Autoradiograms of purified human lymphocytes that were sham-exposed (lane 1) or exposed to influenza virus (lanes 2–7) in the presence of autologous monocytes-macrophages are shown. The lymphocytes were then purified by elutriation before radiolabeling. The lymphocytes had been cocultured with varying proportions of monocytes-macrophages for three days after stimulation with inactivated influenza virus as follows: <0.5% monocytes-macrophages, lanes 2 and 3; 11% monocytes-macrophages, lanes 4 and 5; 26% monocytes-macrophages (equivalent to unseparated PBMC), lanes 1 (control), 6, and 7. Cells were then exposed to infectious influenza virus, followed by purification by elutriation, with separation into resting and proliferating fractions before radiolabeling. As with the other figures, each lane represents lysate from 2 × 10^6^ cells. Lanes 2, 4, and 6 show lysates from small resting lymphocytes (▲ in Figure S3 and Figure 4; >95% G_0_/G_1_). Lanes 3, 5, and 7 show lysates from lymphocytes enriched (>40%) for cells in S + G_2_/M (● in Figure S3 and Figure 4).

Early studies with influenza virus concluded that virus-exposed human PBMC which had been stimulated with the mitogen PHA synthesized virus proteins, whereas unstimulated cells did not [49]. The effect of lymphocyte proliferation, as well as the percentages of cocultured monocytes-macrophages, on susceptibility to influenza virus infection was examined by exposing cells to infectious virus three days after stimulation with mitogen (PHA) or antigen (inactivated influenza virus, such as the representative experiment shown in Figure 4) in the presence of <0.5%, 11%, or 26% (equivalent to unseparated PBMC) monocytes-macrophages (Figure S3). Similar results were obtained whether the proliferating lymphocyte populations were generated in response to PHA or viral antigen stimulation. Each stimulated cell preparation was elutriated to provide fractions containing small, quiescent (>95% G_0_/G_1_) lymphocytes and fractions containing exponentially expanding (activated lymphoblasts; >40% S + G_2_/M) populations (Figure 4). Cells were then pulse-labeled for two hours. There was no viral protein synthesis in either small, resting lymphocytes (lane 2) or activated lymphoblasts (lane 3) exposed to infectious virus in the absence of monocytes-macrophages (Figure 5). After exposure in the presence of intermediate numbers of monocytes-macrophages (11%), viral protein synthesis was not detected in small resting lymphocytes (lane 4), but was easily demonstrated in populations enriched for large, proliferating lymphocytes-lymphoblasts (lane 5). After exposure in the presence of monocytes-macrophages equivalent to those in unseparated PBMC, viral protein synthesis was demonstrated in both small, resting (lane 6) and large, proliferating (lane 7) lymphocyte populations.

### 3.4. Development of Cell Clusters and Expression of Viral Proteins

**Figure 6:**
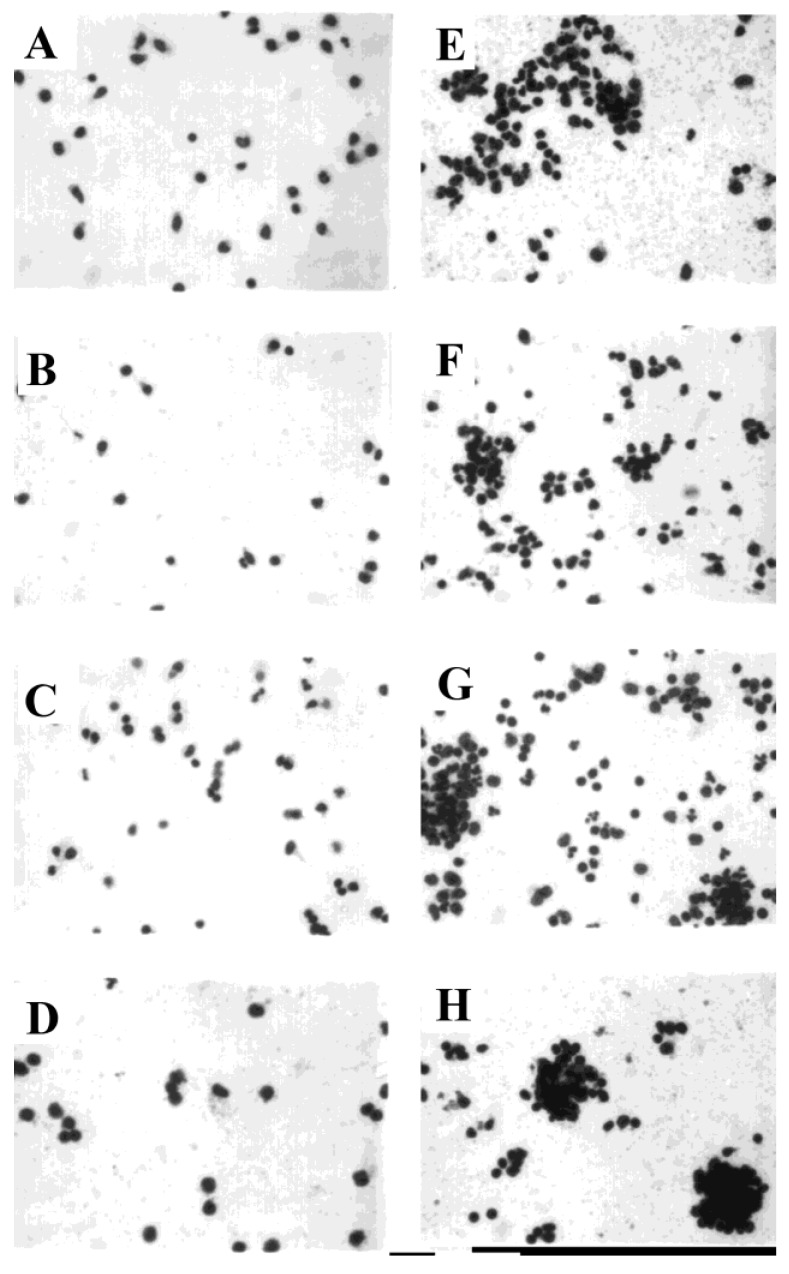
Cell cluster formation by sham-exposed and influenza virus-exposed PBMC (10× image). PBMC were sham-exposed (**A**–**D**) or exposed to influenza virus (**E**–**H**). Cultures were stained and examined by light microscopy at 40× after 1 h (**A**,**E**), 4 h (**B**,**F**), 24 h (**C**,**G**), and 72 h (**D**,**H**). Representative fields are shown.

**Figure 7:**
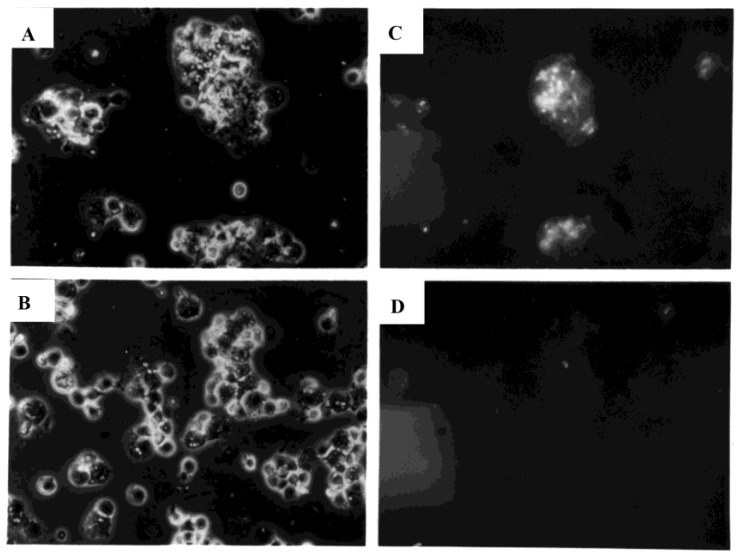
Expression of influenza A hemagglutinin (HA) by PHA-stimulated human PBMC (40× image). Influenza virus (H1N1)-exposed PBMC (upper panels, (**A**,**C**)) and sham-exposed PBMC (lower panels, (**B**,**D**)) were examined by light (left panels) and fluorescence microscopy (right panels; same fields as left) after indirect immunofluorescent staining using influenza H1-specific polyclonal reference antisera. Cells were examined six hours after exposure to virus (MOI = 10).

PBMC were sham-exposed or exposed to influenza virus and stained in situ at early time points after exposure to determine the extent of cell cluster formation, which might provide the microenvironment for monocyte/macrophage-mediated facilitation of lymphocyte infection. Within one hour after exposure to influenza virus, PBMC formed clusters (Figure 6E), with more extensive cell clustering subsequently. In parallel cultures, no anti-hemagglutinin immunofluorescent staining of live, PHA-stimulated cells was seen at six hours (Figure 7, lower panels) after sham exposure to virus, even though the mitogen stimulation induced cell clustering. In contrast, marked hemagglutinin-specific staining was observed at six hours within the virus-exposed cultures, associated with PBMC clusters (Figure 7, upper panels). Little or no fluorescence was associated with solitary cells stained within six hours after exposure to virus.

### 3.5. Uptake of FITC-Conjugated Influenza Virus by Lymphocytes

We then assessed whether monocytes-macrophages were required for the uptake of influenza virus by lymphocytes or were merely necessary to support the transcription and translation of viral gene products after independent lymphocyte uptake of the virus. A series of studies were performed in which purified lymphocytes and PBMC were exposed to FITC-labeled virus to directly examine the uptake of input virus, differentiating external, cell-bound virus from virus that had been internalized by the cells. Cells were analyzed for FITC-derived green fluorescence by flow cytometry, gating on monocytes-macrophages or lymphocytes by light scatter in each arm of an experiment. Then, ethidium bromide was added to ablate the green fluorescence of extracellular fluorescein by quenching and resonance energy transfer. The live cells excluded ethidium bromide, so that internalized FITC-labelled virus continued to be detectable by green fluorescence, whereas external, cell-bound virus was detected by red fluorescence [35].

**Figure 8:**
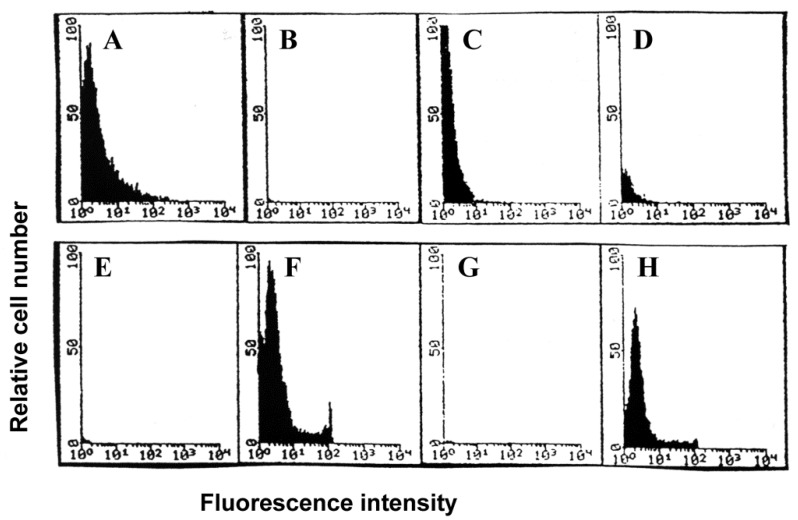
Flow cytometric analysis of binding and internalization of FITC-labeled influenza virus by lymphocytes. Lymphocytes were exposed to virus in the absence (**A**,**E** and **B**,**F**) or presence (**C**,**G** and **D**,**H**) of monocytes-macrophages. Cells (10^4^ per panel) were analyzed 2 h after exposure for green (**A**–**D**) and red (**E**–**H**) fluorescence in the absence (**A**,**E** and **C**,**G**) and presence (**B**,**F** and **D**,**H**) of ethidium bromide. Sequential upper and lower pairs of panels (for example, **A**,**E** and **B**,**F**) show results with the same population of cells analyzed before and after addition of ethidium bromide.

The FITC-labeled virus attached rapidly to the cells in culture. For example, the virus showed incremental binding to monocytes-macrophages (exposed at MOI = 3), and was associated with 1.2% of cells at 2 min, and 3.7%, 16.2%, and 42.0% of cells at 6, 10, and 14 min, respectively, after exposure at 37 °C (with intermediate percentages at intermediate time points). Lymphocytes, whether exposed alone or in the presence of macrophages, adsorbed the labeled virus to their surface and showed green fluorescence 2 h (Figure 8A,C, respectively) or 5 h [50]. When ethidium bromide was added to the cells and the analysis was repeated, the lymphocytes exposed in the absence of macrophages showed a complete shift to red fluorescence (Figure 8F), with no detectable (i.e., green) internalized virus (Figure 8B). In contrast, lymphocytes exposed to virus in the presence of macrophages showed green fluorescence indicative of internalized virus in a portion of the cells (Figure 8D).

**Figure 9:**
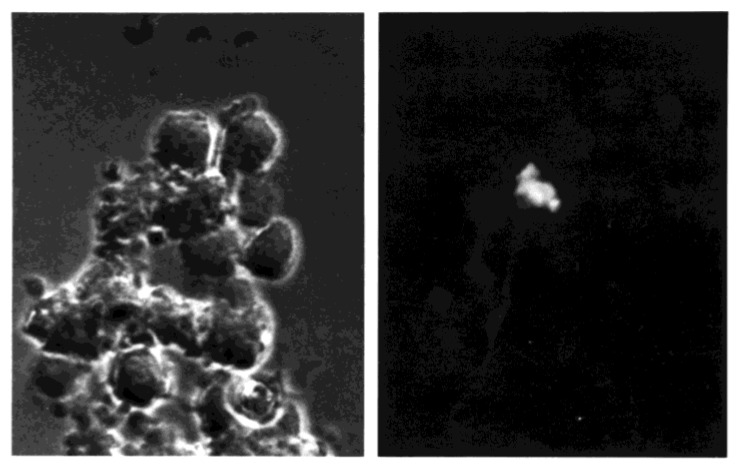
Cell cluster formation by PBMC exposed to FITC-labeled influenza virus (60× image). Cells were examined one hour after exposure to virus (MOI = 3) and examined by light (left panel) and fluorescence microscopy (right panel; same field as left) after addition of ethidium bromide to eliminate detection of green fluorescence associated with bound virus that is external to cell membranes.

**Figure 10:**
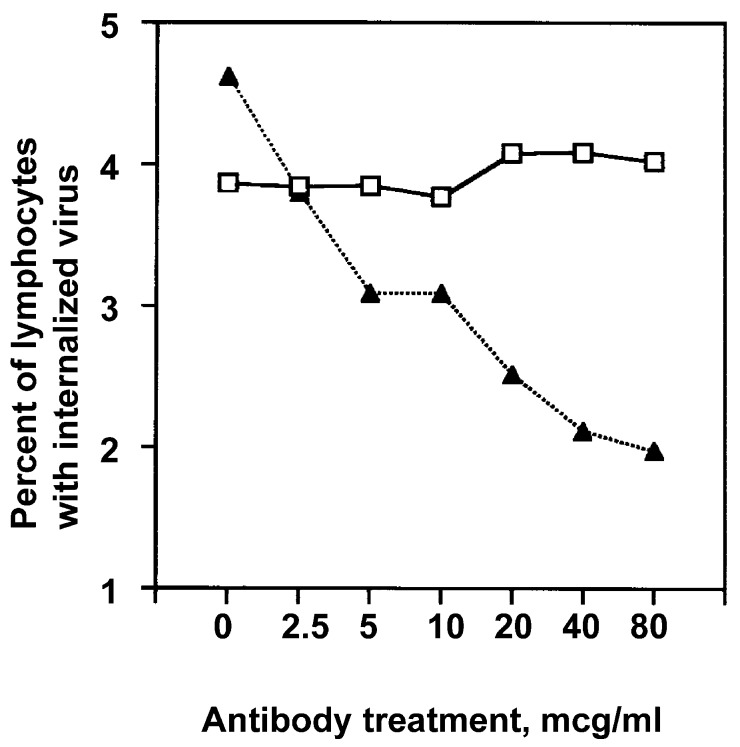
Flow cytometric analysis of internalization of FITC-labeled influenza virus by lymphocytes exposed in the presence of monocytes-macrophages (i.e., as PBMC). PBMC were exposed to virus after pretreatment (▲) or sham pretreatment (isotype control, □) with antibody to ICAM-1. Results are shown for lymphocytes after addition of ethidium bromide to cultures to identify internalized (versus bound) virus.

The internalized virus was detected in association with cell clusters that involved monocytes-macrophages and lymphocytes (Figure 9). The critical role of monocyte-macrophage and lymphocyte clustering for lymphocyte infection by the virus was demonstrated by experiments in which clustering of the unseparated, coexposed cells was prevented by the addition of anti-ICAM-1 antibody. Both naïve and effector T cells require LFA-1–ICAM-1 interaction for immunological synapse formation with antigen-presenting cells such as monocytes-macrophages [51]. Binding of the virus to the lymphocyte cell surface was not affected by either isotype control or anti-ICAM-1 antibody treatment. However, lymphocyte uptake of influenza virus was decreased in a dose-dependent manner when cultures of unseparated PBMC were treated with the anti-ICAM-1 antibody prior to exposure to the virus (Figure 10).

### 3.6. Uptake of FITC-Labeled Virus by Alveolar Lavage Versus Peripheral Blood Lymphocytes

Further studies were undertaken to determine whether the susceptibilities to influenza virus infection of CD4^+^ and CD8^+^ T lymphocytes from peripheral blood are similar to the relative susceptibilities of subsets of T cells that can be recovered by bronchoalveolar lavage. The latter cells might represent a resident lymphocyte population first to be challenged naturally by the virus. Insufficient total numbers of alveolar lavage-derived lymphocytes would be available for PAGE and Northern blot assays, such as described above for peripheral blood-derived lymphocytes. Therefore, we assessed the cellular uptake of FITC-labeled virus.

Lymphocytes in the normal volunteer lavage cell preparations represented a smaller proportion of the total number of cells (14.40% ± 2.19) and the ratios of CD4^+^/CD8^+^ lymphocytes were more variable, as described previously by us and others [52,53]. Overall, however, the mean ratio of CD4^+^/CD8^+^ lavage lymphocytes (1.56 ± 0.68) for all but one subject was relatively similar to that of peripheral blood in our studies described above. One of the normal subjects had a marked preponderance of CD8^+^ lavage lymphocytes (CD4^+^/CD8^+^ ratio = 0.08:1), although the relative proportions of subsets infected were similar to those of the several other subjects (data are presented for all of the subjects studied). Between 1.34 and 4.23% of total peripheral blood-derived T lymphocytes showed uptake of influenza virus. Uptake by CD3^+^, CD4^+^, and CD8^+^ lymphocytes is shown in Table 1. The FITC-labeled virus was taken up by 1.19 to 3.62% of total lavage T lymphocytes using cells from all but one subject; 15.95% of that subject’s lavage lymphocytes internalized the virus. A greater proportion of CD3^+^, CD4^+^, and CD8^+^ alveolar lavage lymphocytes internalized the virus compared with peripheral blood-derived cells (Table 1). Nonetheless, nearly equivalent percentages of CD4^+^ and CD8^+^ lavage lymphocytes were infected. 

### 3.7. Infectious Focus Potential of Influenza Virus-Exposed Lymphocytes

The above and earlier [20] data suggest that monocytes-macrophages could serve as infectious foci for lymphocytes, including both CD4^+^ and CD8^+^ T lymphocytes. Synthesis of viral proteins by the lymphocytes was definite but not intense, likely due to a much lower percentage of cells being infected, as suggested by earlier studies [22] and the above data from studies using FITC-labeled virus. If the macrophages served as infectious foci leading to subsequent viral infection of lymphocytes, those lymphocytes (exposed to virus in the presence of macrophages) might in turn serve as infectious foci for other cells, including virus-permissive cells [48]. Assays of the potential of the CD4^+^ and CD8^+^ lymphocytes to serve as infectious foci for such cells could provide an additional indication of the proportion of subset lymphocytes that were infected, and could thereby provide evidence that the uptake of FITC-labeled virus accurately reflected the internalization of fully infectious virions.

Influenza virus-exposed purified total T lymphocytes and CD4^+^ and CD8^+^ T lymphocytes (exposed to virus in the presence of macrophages before purification) were treated with neuraminidase and tested for ability to serve as infectious foci for virus-permissive MDCK cells. Previous studies by others have shown that neuraminidase treatment removes adherent radiolabeled influenza virus from the cell surface [33,34]. We also showed, using flow cytometric analyses of cells exposed to FITC-labeled virus and treated similarly with neuraminidase, that all detectable virus was removed from the cell surface with such treatment [35].

Between 1 and 3% of total T lymphocytes could serve as infectious foci for the permissive cells after exposure of the leukocytes (as PBMC before separation; Table 2). Protocols performed to compare CD4^+^ and CD8^+^ T lymphocytes required extensive separation procedures after exposure to virus. There was slight variability between donors in the ability of each type of cell to serve as infectious foci. Overall, however, equivalent percentages of CD4^+^ and CD8^+^ T lymphocytes could produce infection of the virus-permissive cells (Table 2). The slight tendency for a greater percentage of CD4^+^ cells to serve as infectious foci (Table 2; ratio of CD4^+^/CD8^+^ = 1.71:1) reflected virtually exactly the CD4^+^/CD8^+^ ratio (1.69:1) of peripheral blood-derived lymphocytes that potentially would be exposed: 45.71% ± 8.71 CD4^+^ and 27.07% ± 7.77 CD8^+^ cells (mean ± SD).

## 4. Discussion

Earlier studies with human cells established that monocytes-macrophages are required for initiation of influenza virus infection of unstimulated (resting) lymphocytes, with infection detected by viral protein synthesis [20]. Monocyte-macrophage-derived supernatant fluids did not effectively substitute for monocytes-macrophages in producing lymphocyte infection. The current studies extend those observations by demonstrating that: (a) monocytes-macrophages are required for the infection of proliferating as well as resting human lymphocytes, although proliferating lymphocytes require fewer monocytes-macrophages to show evidence of infection by synthesis of viral proteins; (b) the transfer of virus is associated with the monocyte-macrophage-lymphocyte clusters occurring during an immune response; (c) both CD4^+^ and CD8^+^ T lymphocytes from both the peripheral blood and alveolar lavage samples are susceptible to influenza virus infection; (d) monocytes-macrophages are required for the initial uptake of the virus by lymphocytes, and are not required merely for the activation of (independently infected) lymphocytes to a state that supports the synthesis of viral proteins; and (e) the virus-infected monocytes-macrophages and CD4^+^ and CD8^+^ lymphocytes could serve as foci for the infection of virus-permissive cells, even though the leukocyte infection itself does not produce progeny virus; the latter finding being demonstrated previously by us and others [12,54]. 

Several comments related to our methods are warranted. Each Figure in the results is representative of at least 4–6 experiments using autologous cells from different donors. Our studies used whole PBMC populations rather than cloned virus-specific cells. Although the approach made it likely that only a small percentage of lymphocytes—the influenza virus-specific cells within the general population [55,56,57]—would cluster with monocytes-macrophages and become infected, the approach was considered to reflect more closely the likely in vivo situation with exposure to a challenging virus. Since we chose to use total normal PBMC preparations rather than cloned cells, we chose elutriation for lymphocyte purification because we needed a large number of highly purified lymphocytes for multiple autologous assays. Alternate methods of purification (for example, using immunomagnetic beads) for several of the experimental protocols would have been unacceptably expensive and would not have increased lymphocyte purity. Purity of the lymphocyte preparations was always confirmed using immunophenotyping and flow cytometric analysis, and the greatest possible percent of contaminating cells at the limits of detection, especially monocytes-macrophages (none detected by analyzing 10,000 cells per preparation; see Figure S1), was shown in initial control experiments not to produce virus-specific bands such as those in lymphocyte lanes exhibited in the results. Previous studies showed that trypsin could not substitute for monocytes-macrophages to produce lymphocyte infection [20], and it was not added to the leukocyte cultures in the current studies.

Monocytes-macrophages were shown to be infected by influenza virus directly in earlier [20] and the current studies. Previous in vitro studies [15,16,17,19] and the current study have shown that direct physical interaction between monocytes-macrophages and T lymphocytes occurs within hours after exposure to either antigens (including infectious influenza virus) or mitogens. The current data suggest that a transfer of influenza virus from monocytes-macrophages to lymphocytes occurs within such clusters. The small percentage of lymphocytes that are infected accounts for the faintness of viral protein bands on electrophoresis autoradiograms compared to the lysates from monocytes-macrophages. It also accounts for the absence of influenza virus-directed shutoff of host cell protein synthesis by autoradiography. The data of the current study correlated well with previously published data regarding the percent of lymphocytes expressing markers of activation and proliferation (HLA-DR, IL-2 receptor, and transferrin receptor) soon after exposure to virus [43]. It is thus appropriate to postulate that the lymphocytes infected in the cluster are at least disproportionately lymphocytes with specificity for influenza virus gene products and thereby also activated. Supporting the concept are results from previous studies which showed that deletion of a small population of virus-reactive lymphocytes eliminated virus-specific immune responses of the remaining PBMC that were tested subsequently [31].

Most recently, others have demonstrated spread of influenza virus to neighboring cells, either Madin–Darby canine kidney cells or a human alveolar cell line (A549), using intercellular connections [58]. Such cell–to-cell-associated infections are not limited to the influenza virus and immunotropic viruses such as the human immunodeficiency virus [59,60,61]. For example, the herpes virus has been reported to replicate in mature dendritic cells, but can only be transferred in a cell–cell contact-dependent manner [62]. Examination of human PBMC interactions with viruses, with attention to events within clusters of monocytes-macrophages and lymphocytes, should lead to further understanding of the immunopathogenesis of the host’s immune response as well as susceptibility to viral infection.

There was good correlation between the results of experiments using uptake of FITC-labeled virus and the experiments assessing cells as infectious foci in regard to the proportions of monocytes-macrophages and CD4^+^ and CD8^+^ lymphocytes that were infected. Analyses for viruses still adherent to cells were negative in the studies assessing the internalization of virus by cells serving as infectious foci, as expected from previous published results using neuraminidase to strip attached but not internalized viruses. The validation of neuraminidase use has been performed previously by us and others using both radiolabeled virus (others’ studies) [33,34] and FITC-labeled viruses (our studies) [35]. Assays for internalization, with and without ethidium bromide, were short in duration to avoid cellular toxicity from the ethidium bromide, and also precluded the confounding aspect of an endocytic decrease in pH over time that would diminish FITC detection. Of course, there is also no reasonable explanation for why endocytic acidification would affect lymphocytes exposed in the absence of monocytes-macrophages, but not lymphocytes exposed in the presence of monocytes-macrophages, even were such effects to exist within the short time frame of the experiments.

Numerous early reports suggested that proliferating lymphocytes are more susceptible to virus infection, including influenza virus, than resting lymphocytes [49,63,64,65,66,67,68,69]. The current study demonstrated an absolute requirement for the presence of monocytes-macrophages for the influenza virus infection of lymphocytes, independent of the proliferative state of the lymphocytes. Large, actively proliferating lymphoblasts as well as small, resting lymphocytes showed no viral protein synthesis after exposure to influenza virus unless exposed in the presence of monocytes-macrophages. However, virus-directed protein synthesis was markedly enhanced in lymphoblast-enriched populations, and a lower percentage of monocytes-macrophages was required to produce evidence of infection than observed with resting lymphocytes, potentially explaining early observations with the influenza virus infection of leukocytes [49]. In the case of polyclonal mitogen stimulation before exposure to virus, lymphocytes of multiple specificities could be recruited into clusters and infected, perhaps contributing to the easier detection of lymphocyte infection with proliferating cells, especially in the setting of limited numbers of monocytes-macrophages.

It is important to note that human PBMC show active proliferation in response to influenza virus with a concomitant suppression of proliferative responses to alternate stimuli, such as a mitogen [38]. Such a reduction in non-influenza-specific proliferation may reduce viral replication after challenge, even though the replication in PBMC is abortive. The benefit of reduced non-influenza-specific proliferation can extend to non-PBMC lung cell populations. For example, mitogenic stimulation can accelerate influenza-induced mortality by increasing susceptibility of alveolar type II cells to infection [70].

The observation that a somewhat greater proportion of both CD4^+^ and CD8^+^ alveolar lavage lymphocytes were infected may be related to the possibility that a greater percentage of lymphocytes that are resident in the lung at any point in time are specific for influenza virus-derived antigens, since the host’s challenge with this virus is respiratory in nature. Other investigators have reported that a large proportion of CD4^+^ and CD8^+^ lymphocytes lavaged from normal subjects exhibit a phenotype associated with long-lived memory cells [52,71]. This is consistent with a potential relationship between the infectivity and virus specificity of the lymphocytes. It is also possible that alveolar lavage lymphocytes are activated to cluster with monocytes-macrophages and become infected based on recent in vivo exposure to alternate antigens. Animal model studies have suggested that influenza antigens can persist in alveoli for weeks [72] to months [73] after exposure, and a high frequency of influenza-specific lung memory cells can be detected months after exposure [74]. Recent studies demonstrated that influenza-specific lung-resident memory T cells are proliferative and polyfunctional [71]. Furthermore, airway-resident CD8^+^ memory T cells can quickly respond to limit early viral replication following secondary influenza virus challenge [75]. Certainly, there was more variability in the uptake of FITC-labeled virus by lavage lymphocytes from experiment to experiment. Within each experiment, however, the uptake of virus by CD4^+^ and CD8^+^ alveolar lavage lymphocytes was similar, ranging from 1 to 2% for CD4^+^ and CD8^+^ cells of some subjects and 6–8% for such cells from others.

Synthesis of viral RNA by CD4^+^ and CD8^+^ cells appeared to be equivalent, but synthesis of viral proteins tended to be greater in CD8^+^ cells than in CD4^+^ cells, each lane having lysate from an equivalent number of cells. This was observed most easily for the nucleoprotein/neuraminidase band on protein gels. The data from the infectious focus and FITC-labeled virus uptake assays combined with the analyses of viral RNA and protein synthesis suggest that CD8^+^ T lymphocytes synthesize viral proteins to a greater extent than CD4^+^ T lymphocytes on a per-cell-infected basis through post-transcriptional processes. Further studies would be needed to fully establish the validity of such an interpretation and to relate such observations to the repertoire of the antiviral immune response.

In conclusion, influenza virus infection commonly resolves with limited morbidity and mortality, as well as with the generation of effective homotypic immunity [12]. Monocytes-macrophages have long been recognized as required accessory cells for influenza antigen-specific responses of lymphocytes, and they also have been shown to play a role in lymphocyte apoptosis after exposure to the influenza virus [76]. The current studies implicate monocytes-macrophages, and the immune cell cluster specifically, in the infection of human lymphocytes by the influenza virus. The events occurring during leukocyte challenge, such as those described in this report, probably affect both the generation and the repertoire of the immune defense against influenza virus infection. Thus, these events are likely to be components of a normal, generally effective, evolutionarily selected, and conserved immune defense. Better delineation of such events may lead to an understanding of the processes underlying the spread within individuals of other viral as well as nonviral intracellular pathogens.

## Figures and Tables

**Table 1:** Percent of influenza virus-infected lymphocytes or lymphocyte subpopulations determined by internalization of FITC-labeled influenza virus.

Source of Cells	Percent of Cells Infected (Mean ± SE)		
	CD3^+^	CD4^+^	CD8^+^
Peripheral blood	2.78 ± 0.60	1.88 ± 0.33	1.78 ± 0.48
Bronchoalveolar lavage	5.96 ± 3.37	4.46 ± 2.00	4.18 ± 0.97

PBMC were exposed to FITC-labeled influenza virus at an MOI of 3, and cells internalizing the virus were identified as described in Materials and Methods one hour after exposure. Results are based on four experiments plus one additional set of determinations for peripheral blood-derived cells. For each individual determination within an experiment, 10,000 cells were analyzed.

**Table 2:** Percent of influenza virus-infected T lymphocytes or lymphocyte subpopulations serving as infectious foci for MDCK cells.

Cells	Experiment						
	1	2	3	4	5	6	Mean ± SE
T lymphocytes	2	3		1			
CD4^+^ lymphocytes	4	1	5	0.4	2	2	2.4 ± 0.7
CD8^+^ lymphocytes	1	1	5	0.1	0.5	1	1.4 ± 0.7

PBMC were exposed to influenza virus at an MOI of 3, and infectivity was determined approximately 7 to 9 h after initial exposure, of which for all but 2 h, the cells were maintained at 4 °C during separation procedures. Serial dilutions of neuraminidase-treated lymphocytes were layered over MDCK cell monolayers and examined after incubation for plaques representing infectious foci, as described in Materials and Methods. Values represent the number of infectious foci per 100 lymphocytes.

## Supplementary Materials

The following are available online at http://www.mdpi.com/1999-4915/10/8/420/s1. Figure S1: Purity of lymphocyte populations after separation procedures. Figure S2: Purity of T-lymphocyte subpopulations after separation procedures. Figure S3: Protocol for stimulation, exposure to virus, and collection and analysis of small, resting lymphocytes and large, proliferating lymphocytes.
