# Supplementary material for: Influenza Virus Infection of Human Lymphocytes Occurs in the Immune Cell Cluster of the Developing Antiviral Response
Source: Viruses. 2018 Aug 10;10(8):420. doi: 10.3390/v10080420 (PMC6115886; doi:10.3390/v10080420)
Supplement: Supplementary file 1 [file viruses-10-00420-s001.pdf]

Supplementary Figures

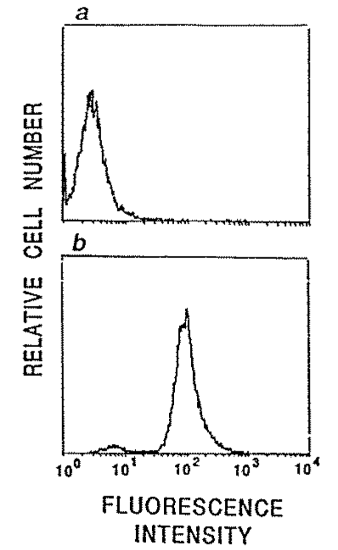

Figure S1. Purity of lymphocyte populations after separation procedures. (*a*) Purified total lymphocytes obtained by elutriation and stained for expression of CD14 (monocyte/macrophage marker). (*b*) Purified T lymphocytes obtained by erythrocyte rosette formation and density gradient sedimentation, and stained for expression of CD3 (T-lymphocyte marker).

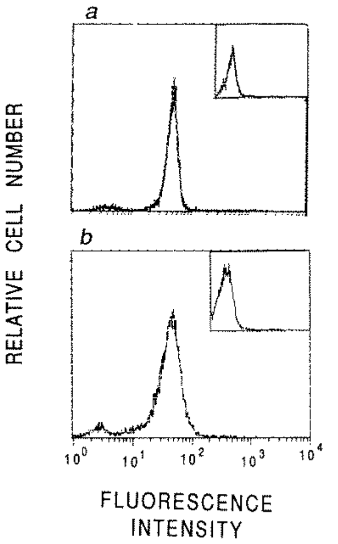

Figure S2. Purity of T-lymphocyte subpopulations after separation procedures. (*a*) Purified lymphocytes obtained by panning and stained for expression of CD4. Inset: Same cells stained for CD14 (monocyte/macrophage marker). (*b*) Purified lymphocytes obtained by panning and stained for expression of CD8. Inset: Same cells stained for CD14.

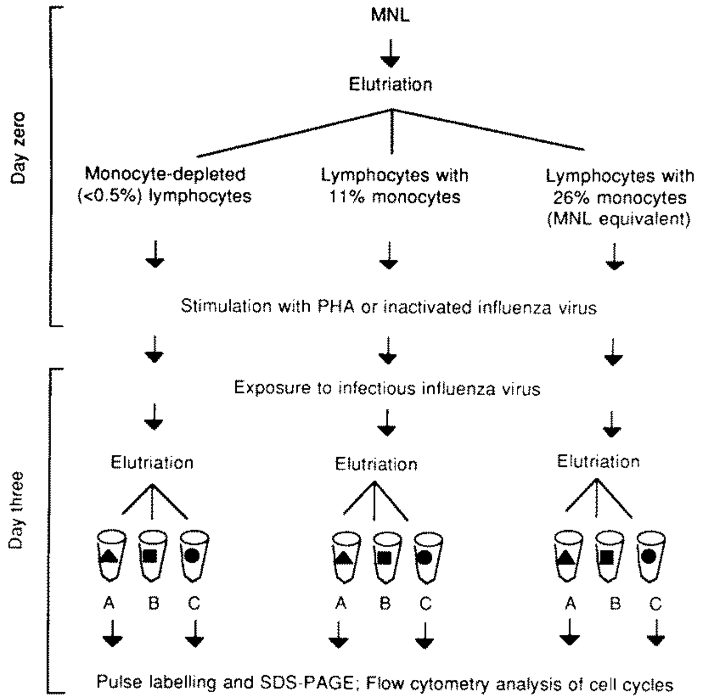

Figure S3. Protocol for stimulation, exposure to virus, and collection and analysis of small, resting lymphocytes (▲) and large, proliferating lymphocytes (●).MNL = peripheral blood mononuclear cells; PHA = the mitogen phytohemagglutinin. The intermediate fractions (■; collected immediately after changing elutriator settings) contained mixed small and large lymphocytes, and were not analyzed further. The symbols correspond to those appearing in Figure 4.
